# Effects of Heat-Treatment and Cold-Rolling on Mechanical Properties and Impact Failure Resistance of New Al 6082 Aluminum Alloy by Continuous Casting Direct Rolling Process

**DOI:** 10.3390/ma17040805

**Published:** 2024-02-07

**Authors:** Jun-Ren Zhao, Fei-Yi Hung, Jian-Hong Chen

**Affiliations:** Department of Materials Science and Engineering, National Cheng Kung University, Tainan 701, Taiwan; a2x346yz03@gmail.com (J.-R.Z.);

**Keywords:** Al 6082 aluminum alloy, continuous casting direct rolling (CCDR), cold rolling, recrystallization, mechanical properties

## Abstract

Al 6082 aluminum alloy has excellent corrosion resistance, strength, and formability. However, owing to the recrystallization effect of a hot working process, coarse grains form easily in this material, which reduces its strength and service life. The novel continuous casting direct rolling (CCDR) method can prevent the deterioration of this material. Thus, we used CCDR Al 6082 aluminum alloy as the research material in this study. By subjecting a CCDR Al 6082 aluminum alloy to heat treatment (T4 and T6) and cold rolling, the influence of recrystallization effect on its mechanical properties and on impact failure resistance were explored. The results demonstrated that the specimen subjected to T4 heat treatment had a higher elongation and that the specimen subjected to T6 heat treatment had a higher strength. After cold rolling, the hardness and strength of the specimens subjected to different heat treatments (coded T4R4 and T6R4) increased because of the work’s hardening effect. Moreover, the elongations of both specimens decreased, but they were higher than the industrial standard (>10%). The strength of specimen T6R4 was higher (up to 400 MPa) than specimen T4R4. Moreover, relative to specimen T4R4, specimen T6R4 had greater tensile and Charpy impact failure toughness.

## 1. Introduction

Al 6082 aluminum alloy was investigated in this study. This material has excellent corrosion resistance, shock absorption, and fatigue resistance. It has been widely used in the manufacture of cars, aircraft, and ships for diverse purposes [1,2,3]. Al 6082 aluminum alloy is a heat-treatable Al-Mg-Si aluminum alloy belonging to the 6xxx series [4,5,6]. The strength can be enhanced through precipitation strengthening by means of solution heat treatment and aging treatment [7,8].

Traditionally, Al 6082 aluminum alloy has been processed using techniques such as casting, die casting, and forging. The strength of cast workpieces is low. Coarse grains are generated and secondary phases such as Al-Fe-Si and Al-Fe-Mn-Cr-Si are precipitated at the grain boundary during the cooling process of cast workpieces. These phenomena decrease the material’s elongation [9,10,11], which must be improved through a homogenization heat treatment [12]. Although forged workpieces have higher strength, toughness, and excellent fatigue and impact resistance properties, coarse grains are generated in them after heat treatment, which reduces their reliability and fracture toughness [13,14,15,16]. These phenomena were also observed in other aluminum alloys, as reported by Lezaack and Simar [17], and Liu et al. [18]. Therefore, novel production process technologies are needed to minimize the above problems.

Continuous casting direct rolling (CCDR), which was employed in this study, is an innovative composite production process involving continuous casting and hot strip rolling [19,20]. The metal structures formed by rapid solidification through CCDR have outstanding material properties and are cost-effective [21]. Moreover, CCDR offers several advantages when applied to the mass production of fasteners such as screws and nuts [22]. Thus far, CCDR has mainly been applied to materials such as steel and copper alloys [22,23,24,25]. Only a few studies have investigated the CCDR process of aluminum alloys. For example, Zhao et al. [22] investigated the cold rolling formability of CCDR 6056 aluminum alloys subjected to different aging temperatures. The over-aging treatment specimens showed the best formability. Huang et al. [26] researched the erosion characteristics of CCDR Al-Si alloy. The specimen was subjected to cold rolling and heat treatment that showed greater wear resistance. Amiri and Saniee [20] studied the influence of the process temperature of CCDR of Al 7075 aluminum alloys. The results indicated that the mechanical properties and grain size were affected by the temperature exponential profile.

There is much literature discussing the evolution of the microstructure and mechanical properties of aluminum alloys after the cold rolling of specimens subjected to heat [27,28]. Some studies have reported the difference between aluminum alloys after heat treatment and those subjected to cold rolling [29,30]. However, there is a lack of discussion on different treatment implementation sequences for cold rolling and heat treatment on one Al alloy material. Thus, the present study builds on our previous research [22,26] to apply the innovative CCDR production process to Al 6082 aluminum alloys. After production, the material was subjected to T4 and T6 heat treatment and cold rolling. The effect of metallurgical dynamics on microstructural characteristics, mechanical properties, and fracture toughness was investigated. The results can provide references for industrial manufacturing applications.

## 2. Experimental Procedure

A CCDR Al 6082 aluminum alloy wire (Φ = 6 mm) was used as the experimental material in this study. The Al 6082 aluminum alloy was manufactured (casting temperature: 760–780 °C, solidification speed: 10^−1^ °C/s) through the CCDR process by Ting Sin Co., Ltd. (Tainan, Taiwan). The composition (in Wt. %) of Al 6082 aluminum alloy is Mg 1.24, Si 1.50, Mn 1.08, Cr 0.62, Ti 0.31, Fe 0.24, Cu 0.09, and Al. The raw material (coded F) was subjected to heat treatment and cold rolling (to simulate cold forging) in different sequences. This study compared the differences in the mechanical properties of the specimens subjected to different sequences of the two treatments. These sequences were such that heat treatment either came before (denoted HC) or after (denoted CH) cold rolling. The specimens with the best mechanical properties were selected for follow-up analyses.

The temperature of the solution for heat treatment was 560 °C [31]. In the present study, this temperature was maintained for 2, 4, and 6 h. According to Chang et al. [32], Al 6082 aluminum alloy reaches a stable state after natural aging for 7 days, whereas 4 h of artificial aging gives rise to the complete precipitation-strengthening effect. Therefore, we denoted natural aging as T4 and artificial aging as T6. In this study, the T4 heat treatment referenced from the literature [31,32] involved solution heat treatment at 560 °C for 2, 4, and 6 h followed by water quenching and natural aging for 7 days (coded T4-2h, T4-4h, and T4-6h, respectively), whereas T6 heat treatment involved solution heat treatment at 560 °C for 2, 4, and 6 h followed by water quenching and artificial aging at 170 °C for 4 h (coded T6-2h, T6-4h, and T6-6h).

In the cold rolling process, the equipment used was our own design and is the same as our previous research article [22,26]. Aluminum alloy wires with Φ = 6 mm were rolled at room temperature to have thicknesses of 5, 4, 3, 2, and 1 mm (coded R5, R4, R3, R2, and R1, respectively, and the corresponding diameter reduction rates were 16.7%, 33.3%, 50%, 66.7%, and 83.3%). When a specimen was found to be cracked after cold rolling, it was not subjected to the subsequent tensile test. The specimen length before and after cold rolling was 90 mm, and the initial circular cross-section (dia: 6 mm) changed into an oblong cross-section. The code name of specimens are listed in Table 1.

Each specimen was ground using SiC sandpaper (progressively finer sandpaper grits were used, beginning with #80 and ending with #4000), and the specimen was then polished using 1 μM and 0.3 μM Al_2_O_3_ solutions and a 0.04 μM SiO_2_ solution. Finally, the polished specimens were etched with Keller’s reagent. An optical microscopy (OM, OLYMPUS BX41M-LED, Tokyo, Japan) was used to observe the microstructure of the specimens. X-ray diffraction (XRD) spectroscopy (Bruker AXS, Karlsruhe, Germany) with a 2θ of 30–90° with 3°/min was used to analyze the phase composition. A Rockwell hardness machine (Mitutoyo, Kawasaki-shi, Japan) was used to perform the Rockwell-F hardness (HRF) test along the rolling direction (RD) plane of the specimens. The test load was 60 kgf, and it was applied using a steel ball head having a diameter of 1/16 in (1.588 mm). The average of 10 measurements was recorded as the experimental data.

**Figure 1:**
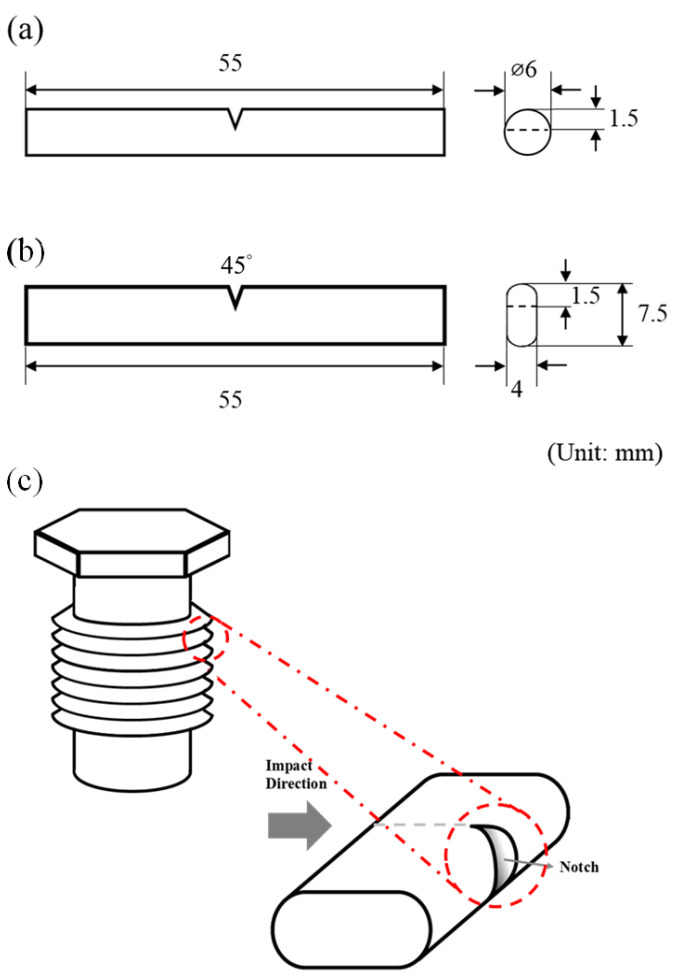
Impact specimen: (**a**) dimensions before cold rolling, (**b**) dimensions after cold rolling, and (**c**) notch of the impact specimen to simulate screw condition.

Tensile tests were performed along the RD by using a universal testing machine (HT-8336, Hung Ta, Taichung, Taiwan) at a tension rate of 1 mm/min. Each experimental data point was the average of five measurements. The tensile fracture surface was observed using a scanning electron microscope (SEM, HITACHI SU-5000, HITACHI, Tokyo, Japan), and the element of the fracture surface was analyzed using an energy dispersive spectrometer (EDS) to clarify the fracture characteristics. An impact tester (Charpy V-notch Impact Test, HUNGTA HT-8041A, Taichung, Taiwan) was used to conduct the impact test. In Charpy impact test, the pendulum weight, pendulum length, and the initial impact angle were 1.54 kgf, 17.61 cm, and 150°, respectively. The size of the impact test bar (specimen dimensions for this study in accordance with ASTM A370 [33]) is illustrated in Figure 1 (a notch in the impact specimen to simulate the actual screw thread condition, as illustrated in Figure 1c). The energy absorbed during the fracture of the test bar was calculated based on the difference between the pendulum angles before and after impact. The resulting value was divided by the cross-sectional area of the fracture surface to obtain the impact value (α). Each experimental data point was the average of 10 Charpy impact measurements. Finally, an SEM was used to observe and analyze the impact section surface.

## 3. Results and Discussion

### 3.1. Comparison of Two Post-Processing Combined Processes

Two post-processing systems, namely, heat treatment and cold rolling, were analyzed in this study. The mechanical properties of the material differ depending on whether heat treatment is applied before cold rolling or after. Therefore, the specimens were divided into two groups: CH (cold rolling first, and then heat treatment) and HC (heat treatment first, and then cold rolling). The influence of different treatment implementation sequences on the mechanical properties of CCDR Al 6082 aluminum alloy was explored and compared.

**Figure 2:**
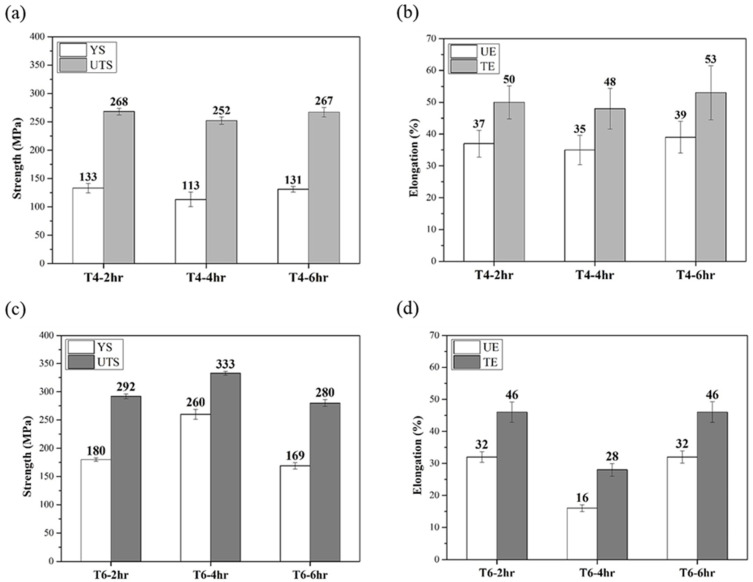
Effects of different heat treatment times on mechanical properties of CCDR Al 6082 aluminum alloy: (**a**) strength of T4, (**b**) elongation of T4, (**c**) strength of T6, and (**d**) elongation of T6 specimens. (YS: yield strength; UTS: ultimate tensile strength; UE: uniform elongation; TE: total elongation).

**Figure 3:**
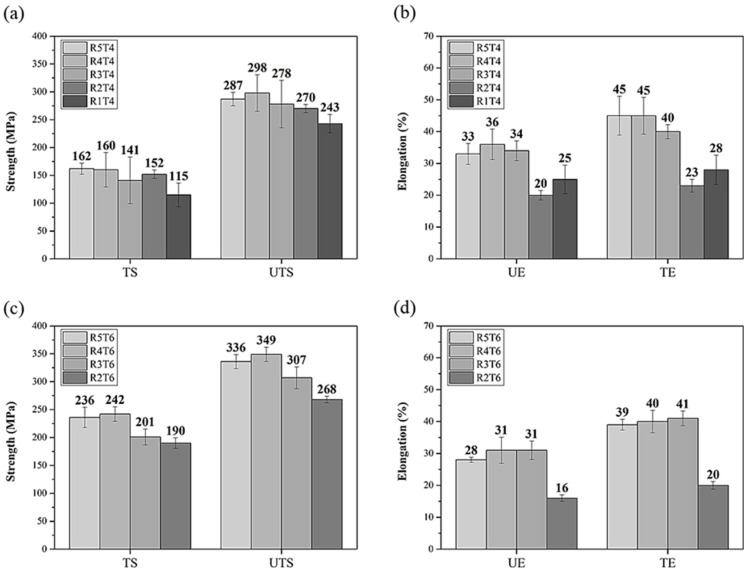
Effect of heat treatment on mechanical properties of CCDR Al 6082 aluminum alloy subjected to different cold rolling conditions: (**a**) strength of T4, (**b**) elongation of T4, (**c**) strength of T6, and (**d**) elongation of T6 specimens. (YS: yield strength; UTS: ultimate tensile strength; UE: uniform elongation; TE: total elongation).

**Figure 4:**
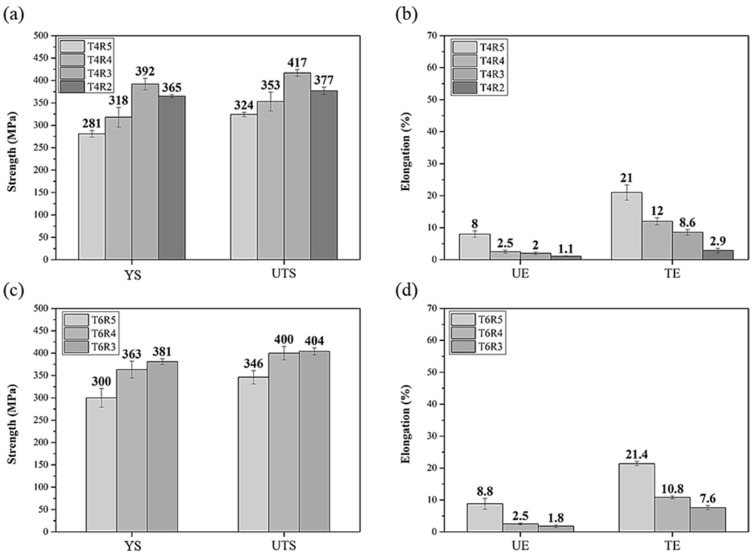
Effects of different cold rolling conditions on mechanical properties of CCDR Al 6082 aluminum alloy after heat treatment: (**a**) strength of T4, (**b**) elongation of T4, (**c**) strength of T6, and (**d**) elongation of T6 specimens. (YS: yield strength; UTS: ultimate tensile strength; UE: uniform elongation; TE: total elongation).

Figure 2 depicts the influence of different solution heat treatment times on the mechanical properties of specimens T4 and T6. The strength of the T4 specimens did not differ between solution heat treatment times (Figure 2a), and a complete solid solution could be obtained after heat treatment for 2 h. In the case of the T6 specimens, 4 h of solution heat treatment time led to a significant decrease in elongation (as shown in Figure 2d). In summary, the optimal solid solution heat treatment times for both types of specimens was 2 h. Furthermore, regardless of the solution heat treatment time, the T6 specimens were stronger and had a marginally lower elongation than the T4 specimens. After the suitable heat treatment conditions (560 °C for 2 h, followed by water quenching, and followed by natural aging for T4 and artificial aging for T6) were verified, the CH and HC specimens subjected to different rolling conditions were compared. Their mechanical properties are depicted in Figure 3 and Figure 4, respectively.

Among the CH specimens, R4T4 and R4T6 were the strongest and had an excellent elongation, as illustrated in Figure 3. Notably, when the reduction rate of cold rolling continued to rise after R4, the mechanical properties of the specimens reduced (especially strength). This indicates that the specimen with the best condition for the reduction rate of cold rolling before heat treatment was R4. The maximum tensile strengths of the HC specimens exceeded 300 MPa (T4R3 and T6R3 were the strongest), and they were higher than those of the CH specimens (Figure 4), but the elongations of the HC specimens were significantly lower. Cold rolling first led to an increase in the internal dislocation density of the CH specimens. Although the dislocations formed a channel, which allowed the atoms of the solid solution to move more easily during the subsequent heat treatment, sufficient annealing and recrystallization were required to ensure the formation of the precipitation strengthening phase; consequently, the precipitation strengthening effect was inadequate [34].

In summary, the HC specimens had a higher strength. The elongation was within the scope of engineering applications until T4R2 and T6R2. When the reduction condition of cold rolling above R3, the elongation was starting to drop below the standard. T4R3 and T6R3 were the strongest specimens, but their elongations were substandard [35]. Among the HC specimens, T4R4 and T6R4 were strong and had higher elongation than the industrial standard (>10%). Therefore, these specimens were selected for follow-up investigations.

### 3.2. Microstructure and Mechanical Properties

**Figure 5:**
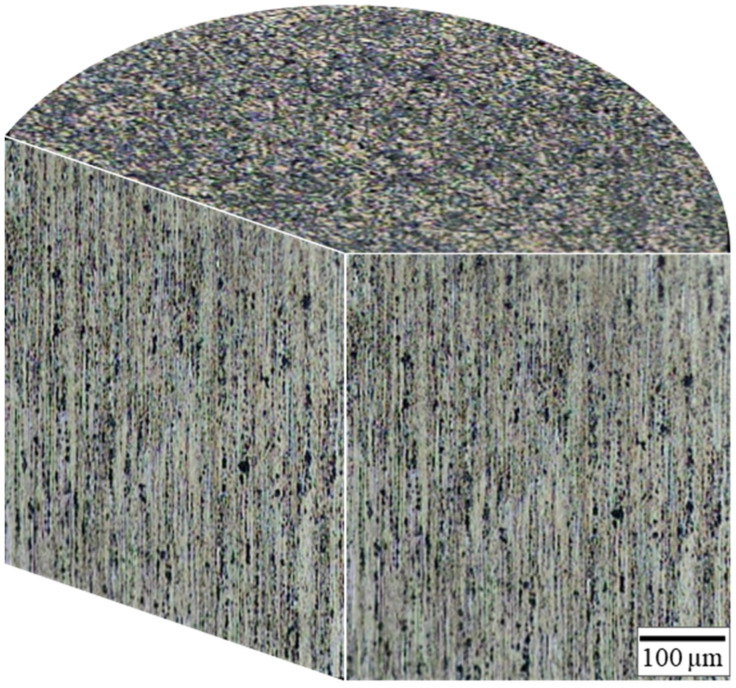
Three-dimensional microstructure of CCDR Al 6082 aluminum alloy.

The three-dimensional (3D) microstructure of the CCDR Al 6082 aluminum alloy wire is depicted in Figure 5. The vertical plane is the RD plane, and the parallel plane is the normal direction (ND) plane. The metal flow lines due to the CCDR process can be observed on the ND plane.

**Figure 6:**
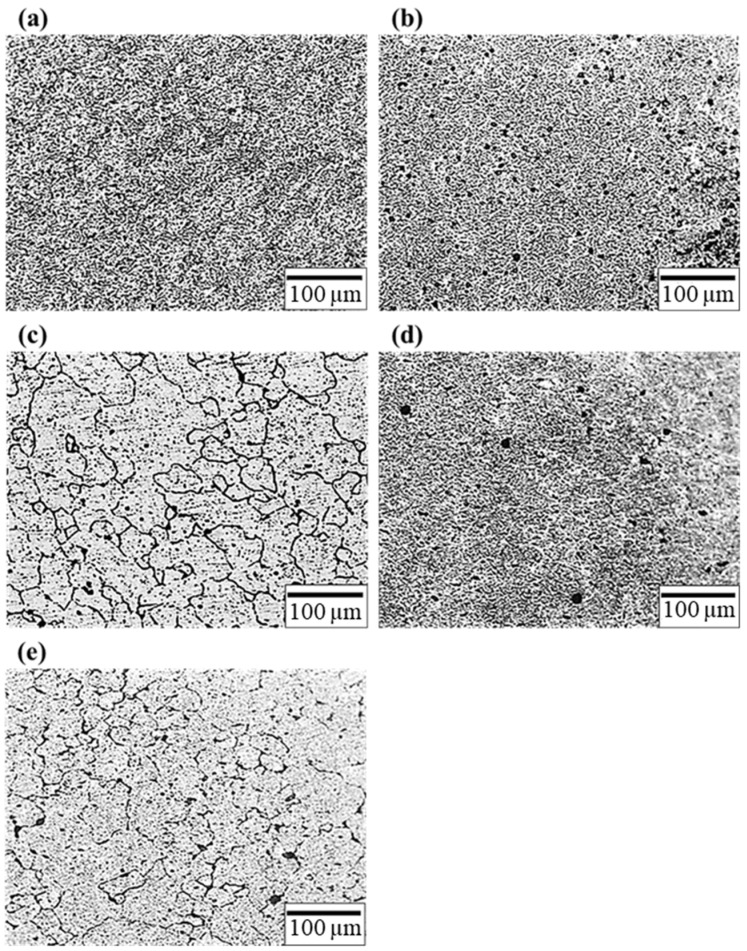
Microstructure of RD plane: (**a**) F, (**b**) T4, (**c**) T6, (**d**) T4R4, and (**e**) T6R4 specimens.

Figure 6 depicts the microstructure of the RD plane of the F, T4, T6, T4R4, and T6R4 specimens. The aluminum matrixes of the T4 and T6 specimens contained black granular precipitates and partially coarse grains, as indicated in Figure 6b,c. The grain boundaries of the specimen after T6 heat treatment were more obvious than those of the specimen after T4. According to the literature [31], the black granular precipitates comprise Mg_2_Si and AlFe(MnCr)Si. The partially coarse grains represent abnormal grain growth due to secondary recrystallization [36,37,38]. The appearance of coarse grains reduces strength and fatigue life in engineering applications. Figure 6d,e depict the microstructures of the RD planes of specimens T4R4 and T6R4, respectively. The coarse grains were eliminated, and the average grain size was significantly smaller due to the cold-working deformation. The strength of these specimens was greatly improved due to the effects of work hardening and grain refinement strengthening.

**Figure 7:**
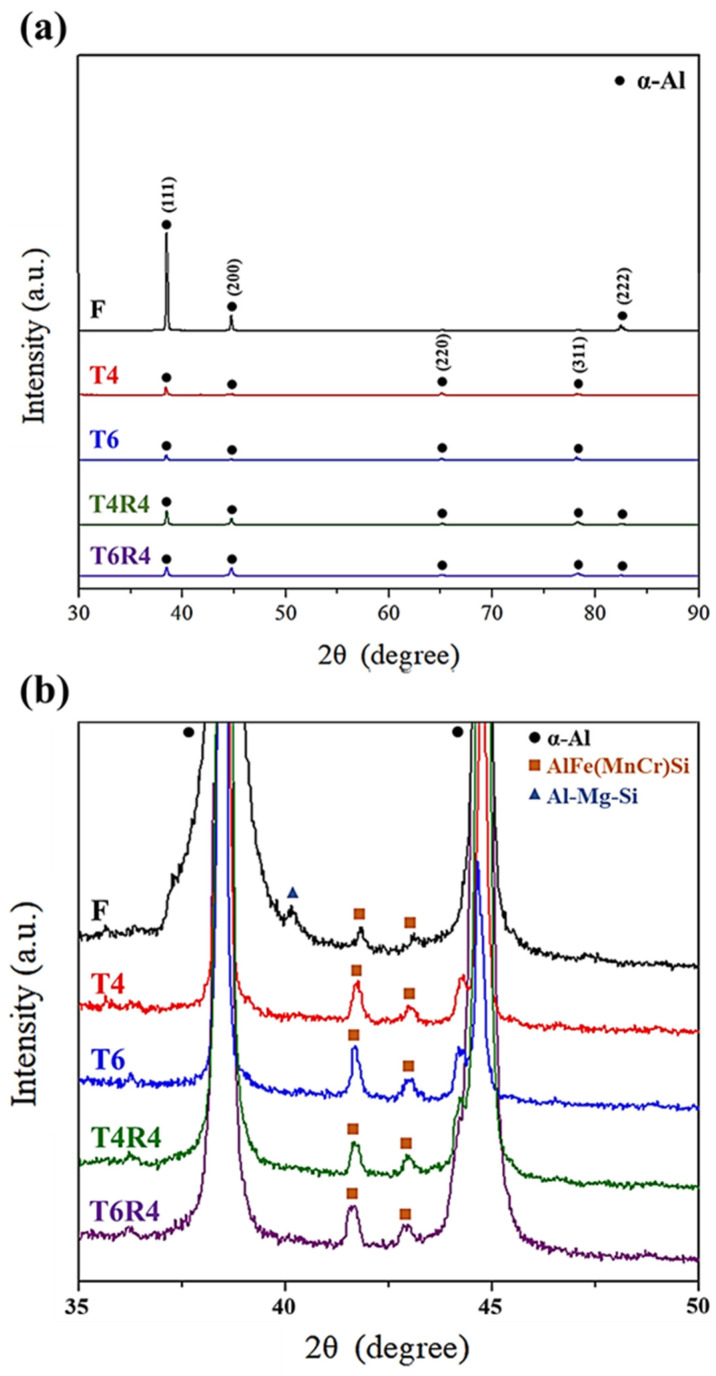
XRD of CCDR 6082 alloy under different heat treatments and cold rolling conditions: (**a**) 30°–90° and (**b**) 35°–50°.

According to the XRD results (Figure 7a), the F specimen had strongly preferred orientation peaks of (111), (200), and (222). After the T4 and T6 heat treatments, the strength of peak (111) decreased, and the peaks of (220) and (311) appeared, whereas that of (222) disappeared. The cold-rolled specimens after the heat treatment showed a peak of (222) appearing again. This means the heat treatments eliminated the metal flow lines caused by the CCDR process to form the equiaxed grains. In addition, the AlFe(MnCr)Si phase existed in all specimens (Figure 7b). After heat treatments (either T4 or T6), the Al-Mg-Si phase dissolved in the matrix. Here, the peak of the AlFe(MnCr)Si phase after the T6 heat treatment was stronger than that of T4 heat treatment. According to the literature [39,40], fine β’’ and β’ precipitates were dispersed throughout the matrix, enhancing the strengthening effect of the T6 heat treatment. These indicate that the precipitation strengthening effect of T6 is more robust than that of T4.

**Figure 8:**
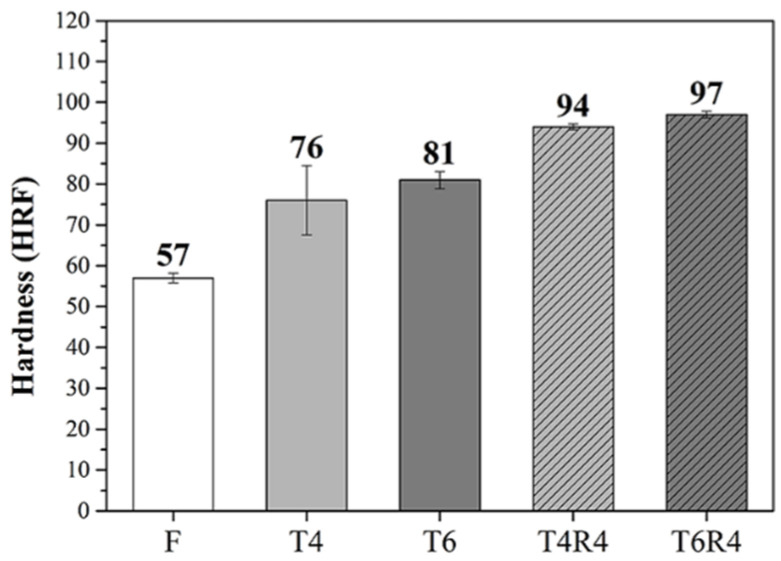
Hardness of CCDR Al 6082 aluminum alloy subjected to different processes.

**Figure 9:**
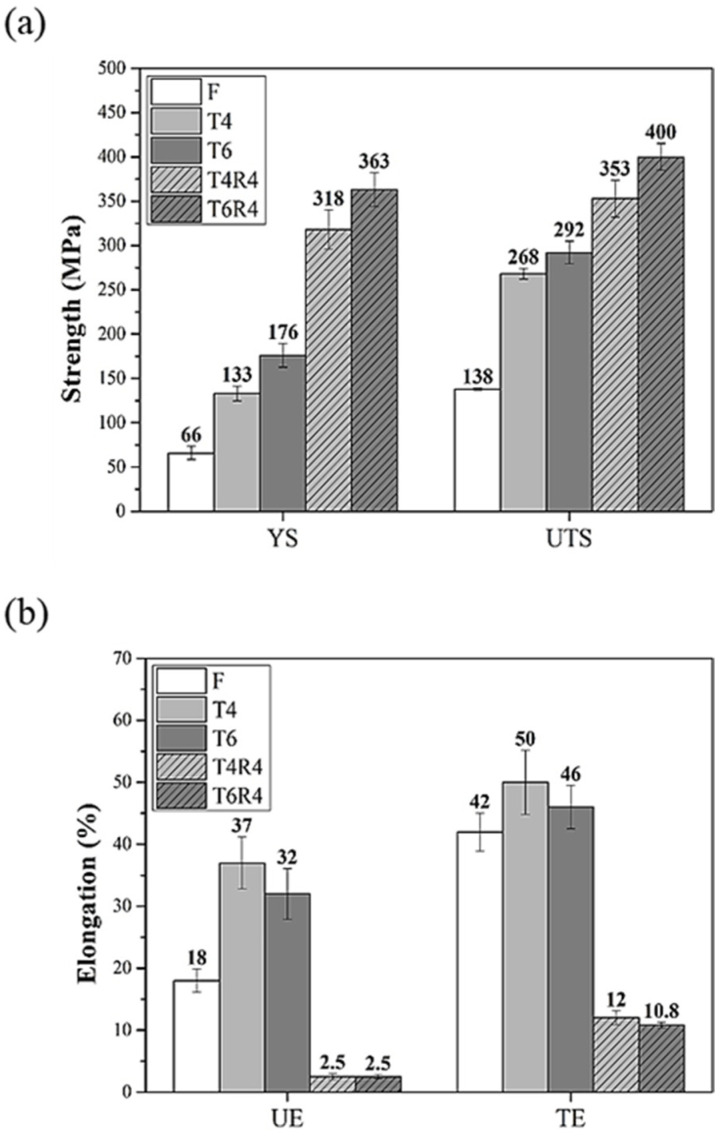
Mechanical properties of CCDR Al 6082 aluminum alloys subjected to different processes: (**a**) strength and (**b**) elongation. (YS: yield strength; UTS: ultimate tensile strength; UE: uniform elongation; TE: total elongation).

Figure 8 illustrates the hardness results of the F, T4, T6, T4R4, and T6R4 specimens. The hardness values of specimens T4 and T6 significantly increased due to the precipitation strengthening of the AlFe(MnCr)Si phase. Because of cold-rolling-induced work hardening, the hardness values of specimens T4R4 and T6R4 further increased to 93 and 97 HRF, respectively. Figure 9 presents the mechanical properties of specimens F, T4, T6, T4R4, and T6R4. Due to aging-heat-treatment-induced precipitation strengthening, the strength and elongation of specimens T4 and T6 increased significantly. Specimen T4 had a more favorable elongation, and specimen T6 was stronger. The internal dislocation density of specimens T4R4 and T6R4 increased after cold rolling, which led to a clear increase in their strengths. Meanwhile, the work hardening effect was significant because of the presence of many dislocation aggregations, which greatly reduced the elongation.

Sonia et al. [41] studied the effect of cryogenic treatment on Al 6082 aluminum alloy. However, the strength of the aluminum alloy is inferior to that of the T4R4 and T6R4 specimens in this study. Zhao et al. [42] applied a solution-forging integrated process to prepare Al 6082 aluminum alloy, which was a continuous process like that in this study. The strength and ductility were approximately 330 MPa and 10.2%, respectively. The mechanical properties of the T4R4 and T6R4 specimens in this study were still better.

**Figure 10:**
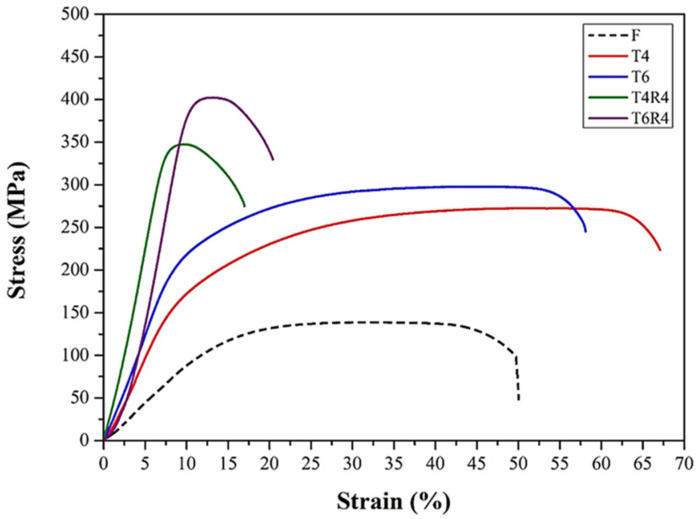
Engineering stress–strain curves of CCDR Al 6082 aluminum alloy subjected to different processes.

According to the engineering stress–strain curve (Figure 10) of specimens T4 and T6, the stress first decreased gently after necking, and the specimens then failed. By contrast, in the cases of specimens T4R4 and T6R4, the stress tended to decrease rapidly after necking. Considering these findings, we deduced that the accumulation of internal dislocations in specimens T4R4 and T6R4 with high dislocation densities after necking induced a readily apparent cold working effect and facilitated crack propagation. The internal dislocation densities of specimens T4 and T6 were lower; cracks in these specimens were initiated from the crystallized phase Mg_2_Si and AlFe(MnCr)Si. Therefore, the deformation resistance bearing the maximum tensile strength of specimens T4 and T6 was better than its counterparts.

**Figure 11:**
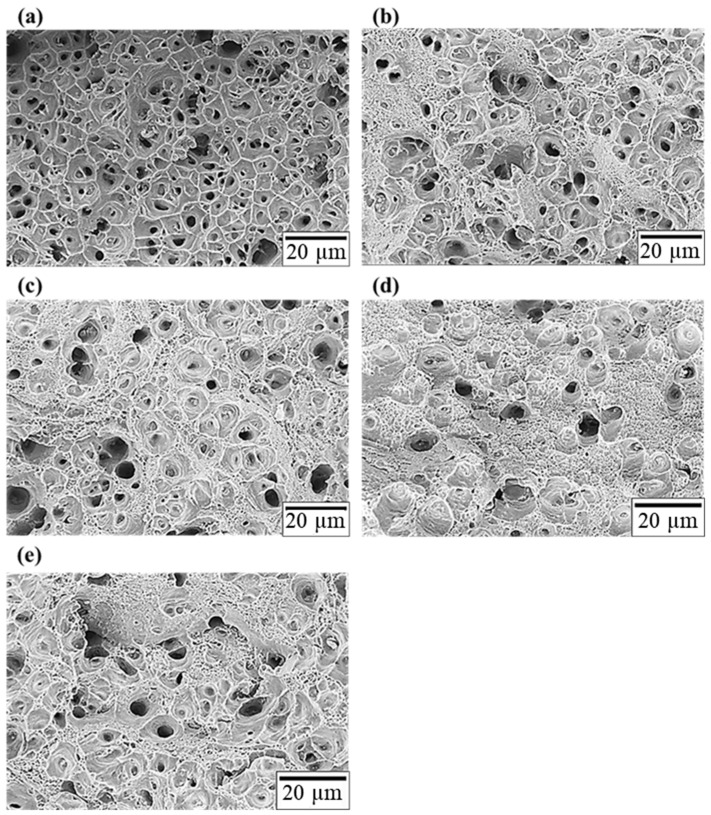
Morphologies of tensile fracture surfaces: (**a**) F, (**b**) T4, (**c**) T6, (**d**) T4R4, and (**e**) T6R4 specimens.

Figure 11 depicts the tensile fracture surfaces of all specimens. The fracture surface of specimen F exhibits a uniform dimpled pattern because of the presence of a coarse crystal phase in the structure, indicating that it is a ductile fracture, as shown in Figure 9 and Figure 11a. The fracture surfaces of T4 and T6 exhibit small and big dimple patterns in Figure 11b,c, respectively. The large area of these big and deep dimples indicates excellent elongation. The fracture morphologies of specimens T4R4 and T6R4 with significantly weaker elongation exhibited more shallow dimples. The flat areas on these surfaces were greater than the dimpled areas, as illustrated in Figure 11d,e, respectively. There were no significant differences between the tensile fracture surfaces of T4 and T6 or T4R4 and T6R4, where all are ductile fractures.

**Figure 12:**
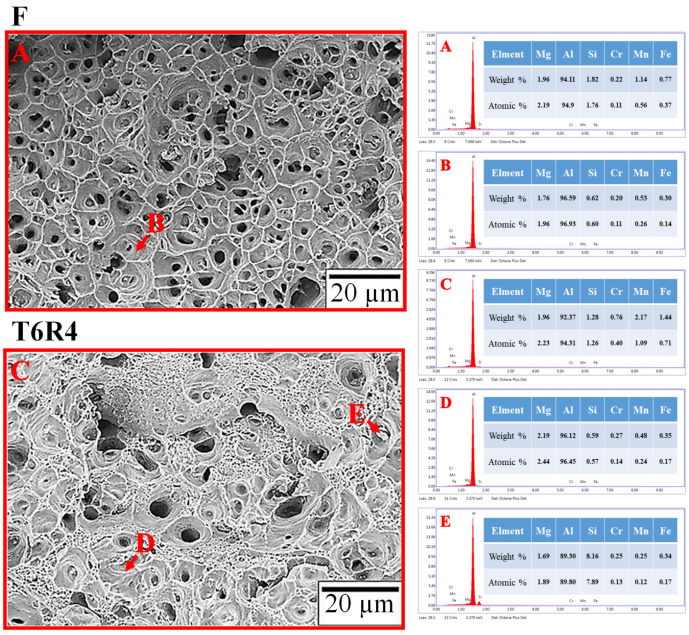
EDS analysis of fracture surface of F and T6R4. (**A**): all F specimen; (**B**): the dimple of the F specimen; (**C**): all T6R4 specimen; (**D**): the dimple of the T6R4 specimen; (**E**): the particle in the dimple of the T6R4 specimen.

According to the EDS results (Figure 12 and Table 2), the content of the Mn, Cr, and Fe elements of the T6R4 specimen was higher than that of the F specimen. For the element distribution of the dimples at the fracture surfaces, it can be observed that the elements distribution of the dimples (B and D) is similar in both the F and T6R4 specimens. The particle in the dimple (E) shows a higher Si element content than the dimples, inferring that the particle is the AlFe(MnCr)Si intermetallic compound [43]. This means the T6R4 specimen was fractured from the crystallized phase and subsequently transferred to the interface between the matrix and particles [37].

### 3.3. Tensile Toughness, Charpy Impact Toughness, and Elasticity Modulus

The toughness of the specimens was evaluated in terms of the amount of energy absorbed in the tensile and impact tests. The absorbed energies were obtained from the area under the stress–strain curve and the impact test results, respectively, as summarized in Table 3. This subsection details the tensile fracture toughness and Charpy impact toughness of specimen T4R4 and the stronger specimen T6R4.

**Figure 13:**
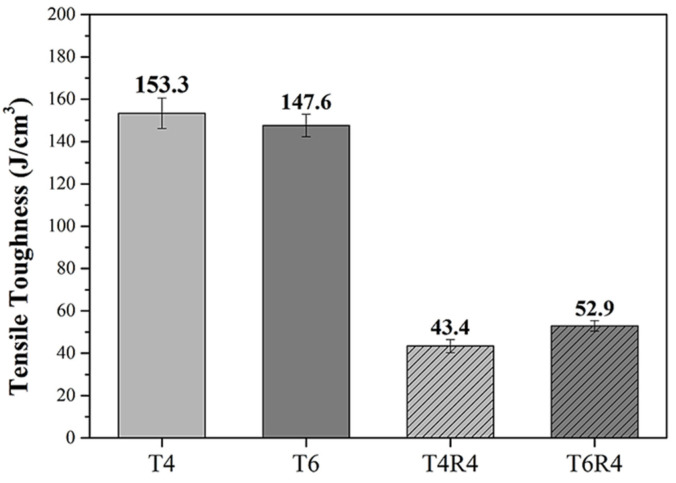
Tensile toughness of specimens T4 and T6 before and after cold rolling.

The tensile fracture toughness was determined from the area under the stress–strain curve (Figure 10). The calculated tensile fracture toughness is depicted in Figure 13. Tensile fracture and Charpy impact toughness did not significantly differ between specimens T4R4 and T6R4. However, both toughness values were marginally higher in specimen T6R4 than in specimen T4R4 (as summarized in Table 3). Furthermore, according to Table 4, it is evident that regardless of the post-processing combination employed, there is a significant improvement in the modulus of elasticity. Notably, T4R4 and T6R4, which undergo heat treatment followed by cold rolling, exhibit the highest values.

**Figure 14:**
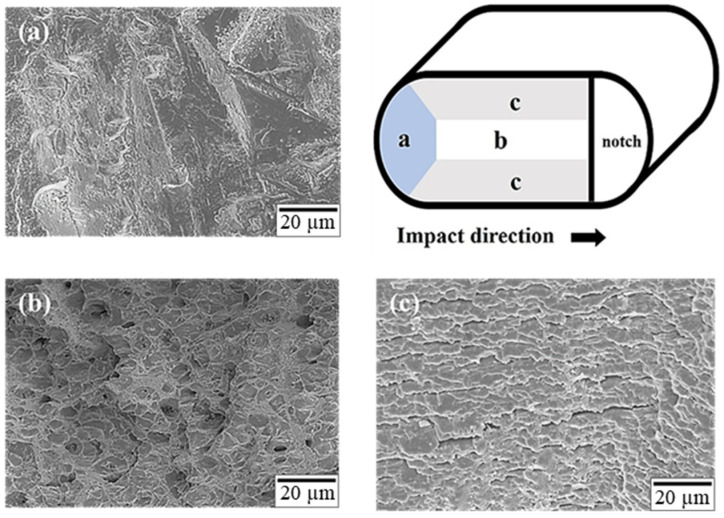
Microstructure of the impact fracture surface of specimen T4R4: (**a**) region a, (**b**) region b, and (**c**) region c.

**Figure 15:**
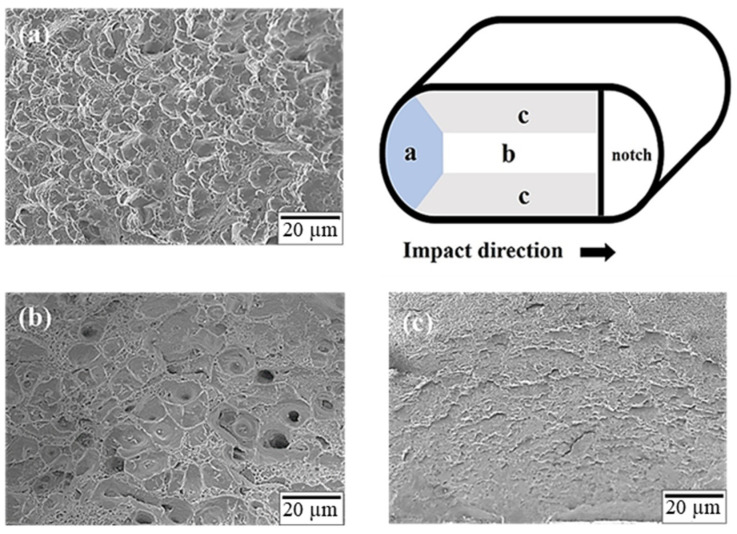
Microstructure of the impact fracture surface of specimen T6R4: (**a**) region a, (**b**) region b, and (**c**) region c.

The notch at the center of the impact specimen was created to simulate the shape of an actual screw, as depicted in Figure 1c. After the completion of the impact test, the fracture surface was observed. The impact fracture surfaces of specimens T4R4 and T6R4 are depicted in Figure 14 and Figure 15, respectively. The impact fracture surface was divided into three regions, namely, a, b, and c, for observation (excluding the grooved area). The colored part represents the area of effective influence.

As depicted in Figure 1a, the initial impact zone (region a) of specimen T4R4 was characterized by plastic deformation mainly due to slip, and the zone had a flatter stepped morphology. By contrast, the initial impact zone of specimen T6R4, depicted in Figure 15a, presented with a shallow dimpled structure. When the impact force was transmitted to the center of the specimen, the fracture had a dimpled surface, as illustrated in Figure 14b and Figure 15b. The aforementioned type is typical ductile fracture morphologies. Both sides of the two specimens exhibited shallow dimpled structures, as depicted in Figure 14c and Figure 15c. According to the literature [44], a greater prevalence of the area with the dimpled structures and the presence of larger dimples are indicative of a higher Charpy impact toughness. The region of specimen T4R4 presented with a brittle fracture morphology, whereas that of specimen T6R4 presented with a dimple morphology. Moreover, the dimples on specimen T6R4 were larger, as evident from a comparison of Figure 14b and Figure 15b. Therefore, the impact fracture resistance of specimen T6R4 was marginally higher than that of specimen T4R4.

**Figure 16:**
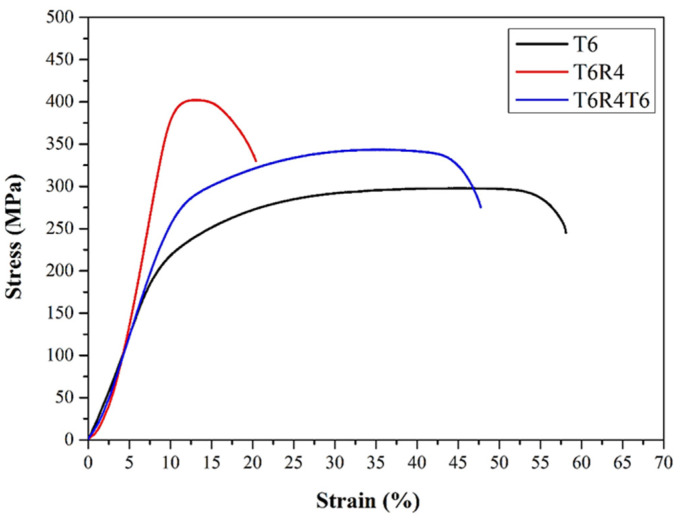
Engineering stress–strain curves of specimens T6, T6R4, and T6R4T6.

**Figure 17:**
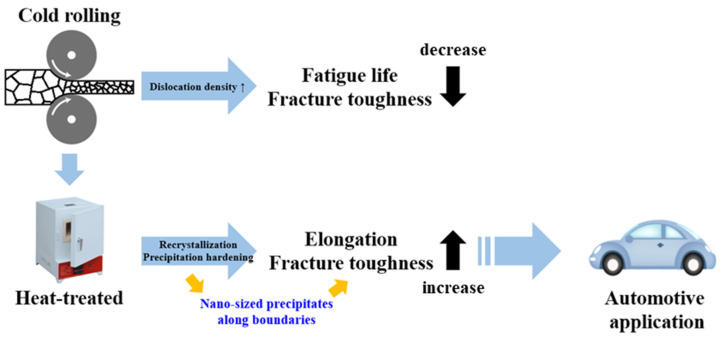
Schematic diagram of the precipitation mechanism and application of specimen T6R4T6.

These results indicated that cold rolling increased the internal dislocation density, resulting in secondary recrystallization. This phenomenon reduced the fatigue life and fracture toughness of the material [45]. To mitigate these adverse effects, specimen T6R4 was subjected to T6 heat treatment. The crystallized phase was dissolved back into the matrix to form a more uniform crystallized phase. The strain due to cold rolling led to recrystallization behaviors during T6 heat treatment. Thus, the dislocation aggregation decreased, the grains were refined, and the nano-sized precipitates were formed along boundaries [46], resulting in the restoration of elongation and fracture toughness (Figure 16). The elongation of specimen T6R4T6 increased, and its tensile fracture toughness (area under the curve) improved, in reference to the automotive industry manufacturing applications (as shown in Figure 17).

### 3.4. Metallurgical Mechanism of CCDR Al Alloy

According to Zhao et al. [42], the use of a solution-forging integrated process can suppress abnormal grain growth. Therefore, it can be inferred that a similar CCDR process in this study can effectively prevent abnormal grain growth. Additionally, adding Mn element to the Al-Mg-Si alloy can enhance recrystallization resistance, thereby improving its strength [47].

Bo et al. [48] studied the effects of cold rolling and heat treatment on AA 5052 Al alloy. The results showed that the strength increased as the rolling reduction increased, which is similar to the results in Figure 4. Notably, the highest strength was when the reduction was 66.7% in this study. For the reasons mentioned above, 6XXX Al alloys have a cold rolling limitation, while 5XXX Al alloys can withstand reductions of up to 90% [28,46,49].

Kumar et al. [46] and Kang et al. [50] both explored the effects of post-rolling heat treatment on recrystallization and mechanical properties. Solid solution heat treatment after rolling induces recrystallization and the grain coarsening, leading to the disappearance of the rolling effect and a decline in mechanical properties. Moreover, cracks were noted in the cold rolling reduction of the 6056 Al alloy without heat treatment when the reduction was approximately 73% in previous research [22]. Clearly, heat treatment is essential for 6XXX aluminum alloy before cold rolling.

The rolling process employed in this study was conducted at room temperature, leading to dynamic recovery. This results in a decrease in low-angle grain boundaries (LAGB) and an increase in high-angle grain boundaries (HAGB), making the material susceptible to softening in subsequent heat treatments [51]. The rise in HAGB content suggests an improvement in the elongation of the specimen [52]. This is the reason why the strength of T6R4T6 in this study was not higher than that of T6R4 (only a 33% increase in this study). It is also consistent with the findings that the test pieces rolled first and then heat-treated exhibit higher ductility. The strength mechanism of the mechanical properties of 6056 and 6082 Al alloys with three-stage processes (heat treatment → cold rolling → heat treatment) is the same.

## 4. Conclusions

In this study, the specimens underwent two sets of treatments: CH and HC. The research aimed to investigate the impact of different diameter reduction rates and heat treatment conditions on CCDR Al 6082 aluminum alloy. Future research directions could involve applying CCDR Al 6082 aluminum alloy in cold forging processes or screw products and exploring the fatigue properties and material failure toughness.

(1) The strength of specimen T6 was notably higher than that of specimen T4 under the same solution heat treatment conditions. This discrepancy is attributed to the weaker precipitation strengthening effect in specimen T4 compared to T6, resulting in lower deformation resistance and higher elongation in specimen T4.
(2) Solution heat treatment after rolling induces recrystallization and grain coarsening, leading to the disappearance of the rolling effect and a decrease in strength. Conversely, heat treatment followed by rolling enhances strength. For diameter reductions of up to 33.3% (R4) due to cold rolling after heat treatment, the elongation remains within the engineering application range (TE > 10%), while maintaining adequate mechanical strength.
(3) The elastic modulus of specimens T4R4 and T6R4 increased from the original 2.13 GPa and 2.67 GPa to 4.98 GPa and 5.96 GPa, respectively. However, the elongation decreased from 10% to 12% after cold rolling, and the toughness also decreased by 71.7% and 64.2%, respectively, after stretching. Despite this, the strength, tensile toughness, and Charpy impact toughness of specimen T6R4 were slightly higher than those of specimen T4R4.
(4) The CCDR process prevented abnormal grain growth. Initially, precipitation strengthening occurred with T4 and T6 heat treatments. Subsequently, the cold rolling process was implemented, promoting the accumulation of dislocations and significantly enhancing the hardening effect, further increasing the strength but reducing the plasticity. Finally, heat treatment was performed to soften the material and restore the ductility reduced by rolling.

## Figures and Tables

**Table 1:** Specimens’ coded names corresponding to processes.

Coded	Treatment Condition
F	Raw material
T4-2h	560 °C 2 h/water quenching → natural aging 7 days
T4-4h	560 °C 4 h/water quenching → natural aging 7 days
T4-6h	560 °C 6 h/water quenching → natural aging 7 days
T6-2h	560 °C 2 h/water quenching → 170 °C 4 h/air quenching
T6-4h	560 °C 4 h/water quenching → 170 °C 4 h/air quenching
T6-6h	560 °C 6 h/water quenching → 170 °C r 4 h/air quenching
R5	Thicknesses of 6 mm were rolled to 5 mm
R4	Thicknesses of 6 mm were rolled to 4 mm
R3	Thicknesses of 6 mm were rolled to 3 mm
R2	Thicknesses of 6 mm were rolled to 2 mm
R1	Thicknesses of 6 mm were rolled to 1 mm
R5T4	Thicknesses of 6 mm were rolled to 5 mm + 560 °C 2 h/water quenching → natural aging 7 days
R4T4	Thicknesses of 6 mm were rolled to 4 mm + 560 °C 2 h/water quenching → natural aging 7 days
R3T4	Thicknesses of 6 mm were rolled to 3 mm + 560 °C 2 h/water quenching → natural aging 7 days
R2T4	Thicknesses of 6 mm were rolled to 2 mm + 560 °C 2 h/water quenching → natural aging 7 days
R1T4	Thicknesses of 6 mm were rolled to 1 mm + 560 °C 2 h/water quenching → natural aging 7 days
R5T6	Thicknesses of 6 mm were rolled to 5 mm + 560 °C 2 h/water quenching →170 °C 4 h/air quenching
R4T6	Thicknesses of 6 mm were rolled to 4 mm + 560 °C 2 h/water quenching →170 °C 4 h/air quenching
R3T6	Thicknesses of 6 mm were rolled to 3 mm + 560 °C 2 h/water quenching →170 °C 4 h/air quenching
R2T6	Thicknesses of 6 mm were rolled to 2 mm + 560 °C 2 h/water quenching →170 °C 4 h/air quenching
R1T6	Thicknesses of 6 mm were rolled to 1 mm + 560 °C 2 h/water quenching →170 °C 4 h/air quenching
T4R5	560 °C 2 h/water quenching → natural aging 7 days + Thicknesses of 6 mm were rolled to 5 mm
T4R4	560 °C 2 h/water quenching → natural aging 7 days + Thicknesses of 6 mm were rolled to 4 mm
T4R3	560 °C 2 h/water quenching → natural aging 7 days + Thicknesses of 6 mm were rolled to 3 mm
T4R2	560 °C 2 h/water quenching → natural aging 7 days + Thicknesses of 6 mm were rolled to 2 mm
T4R1	560 °C 2 h/water quenching → natural aging 7 days + Thicknesses of 6 mm were rolled to 1 mm
T6R5	560 °C 2 h/water quenching →170 °C 4 h/air quenching + Thicknesses of 6 mm were rolled to 5 mm
T6R4	560 °C 2 h/water quenching →170 °C 4 h/air quenching + Thicknesses of 6 mm were rolled to 4 mm
T6R3	560 °C 2 h/water quenching →170 °C 4 h/air quenching + Thicknesses of 6 mm were rolled to 3 mm
T6R2	560 °C 2 h/water quenching →170 °C 4 h/air quenching + Thicknesses of 6 mm were rolled to 2 mm
T6R1	560 °C 2 h/water quenching →170 °C 4 h/air quenching + Thicknesses of 6 mm were rolled to 1 mm
T6R4T6	560 °C 2 h/water quenching →170 °C 4 h/air quenching + Thicknesses of 6 mm were rolled to 4 mm + 360 °C 2 h/water quenching →170 °C 4 h/air quenching

**Table 2:** Energy dispersive spectrometer (EDS) analysis results of fracture specimens, as shown in Figure 13.

Locations	A		B		C		D		E	
Elements	Wt. %	At. %	Wt. %	At. %	Wt. %	At. %	Wt. %	At. %	Wt. %	At. %
Mg	1.96	2.19	1.76	1.96	1.96	2.23	2.19	2.44	1.69	1.89
Al	94.11	94.99	96.59	96.93	92.37	94.31	96.12	96.45	89.30	89.80
Si	1.82	1.76	0.62	0.60	1.28	1.26	0.59	0.57	8.16	7.89
Cr	0.22	0.11	0.20	0.11	0.76	0.40	0.27	0.14	0.25	0.13
Mn	1.14	0.56	0.53	0.26	2.17	1.09	0.48	0.24	0.25	0.12
Fe	0.77	0.37	0.14	0.14	1.44	0.81	0.38	0.17	0.34	0.17

**Table 3:** Comparison of tensile fracture toughness and Charpy impact toughness.

Specimen	T4R4	T6R4
Tensile Toughness (J/cm^3^)	43.4 ± 3.1	52.9 ± 2.5
Charpy Impact Toughness (J/cm^2^)	11.6 ± 1.5	12.3 ± 2.3

**Table 4:** Comparison of modulus of elasticity of Al 6082 aluminum alloy processed through different processes.

Specimen	F	T4	T6	T4R4	R4T4	T6R4	R4T6
E (GPa)	0.87	2.13	2.67	4.98	4.46	5.16	4.52

## Data Availability

Data are contained within the article.
